# Supplementary material for: Influence of the TorD signal peptide chaperone on Tat-dependent protein translocation
Source: PLoS One. 2021 Sep 9;16(9):e0256715. doi: 10.1371/journal.pone.0256715 (PMC8428690; doi:10.1371/journal.pone.0256715)
Supplement: S1 Fig — The signal peptides of TorA and SufI are underlined, the TEV sequence is identified in blue, the QSV tag is identified in green, and other additions/linkers/mutations are indicated in red. (PDF) [file pone.0256715.s001.pdf]

TorD-H6
(plasmid pTorD-H6; *Addgene #169039*):

MVTTLTAQQIACVYAWLAQLFSRELDDEQLTQIASAQMAEWFSLLKSEPPLTAAVNELENRIATLTVRDD
ARLELAADFCGLFLMTDKQAALPYASAYKQDEQEIKRLLVEAGMETSGNFNEPADHLAIYLELLSHLHFS
LGEGTVPARRIDSLRQKTLTALWQWLPEFVARCRQYDSFGFYAALSQLLLVLVECDHQNRSHHHHHH

H6-spTorA-mCherry
(plasmid pH6-spTorA-mCherry; *Addgene #169040*):

MAHHHHHHNNNDLFQASRRRFLAQLGGLTVAGMLGPSLLTPRRATAAQAASIDSKGEEDNMAIIKEFMRF
KVHMEGSVNGHEFEIEGEGEGRPYEGTQTAKLKVTKGGPLPFAWDILSPQFMYGSKAYVKHPADIPDYLK
LSFPEGFKWERVMNFEDGGVVTVTQDSSLQDGEFIYKVKLRGTNFPSDGPVMQKKTMGWEASSERMYPED
GALKGEIKQRLKLKDGGHYDAEVKTTYKAKKPVQLPGAYNVNIKLDITSHNEDYTIVEQYERAEGRHSTG
GMDELYK

H6-spTorA-GFP
(plasmid pH6-TEV-spTorA-GFP(C); *Addgene #169041*):

MAHHHHHHENLYFQGGANNNDLFQASRRRFLAQLGGLTVAGMLGPSLLTPRRATAAQAARKGEELFTGVV
PILVELDGDVNGHKFSVSGEGEGDATYGKLTLKFICTTGKLPVPWPTLVTTFGYGVQCFARYPDHMKRHD
FFKSAMPEGYVQERTIFFKDDGNYKTRAEVKFEGDTLVNRIELKGIDFKEDGNILGHKLEYNYNHHNVYI
TADKQKNGIKANFKIRHNIEDGSVQLADHYQQNTPIGDGPVLLPDNHYLDTHSALSKDPNEKRDHMVLLE
FVTAAGITHGMDELYKLEC

spTorA-GFP-H6C
(plasmid p-spTorA-GFP-H6C; *Addgene #168517*):

MANNNDLFQASRRRFLAQLGGLTVAGMLGPSLLTPRRATAAQAARKGEELFTGVVPILVELDGDVNGHKF
SVSGEGEGDATYGKLTLKFICTTGKLPVPWPTLVTTFGYGVQCFARYPDHMKRHDFFKSAMPEGYVQERT
IFFKDDGNYKTRAEVKFEGDTLVNRIELKGIDFKEDGNILGHKLEYNYNHHNVYITADKQKNGIKANFKI
RHNIEDGSVQLADHYQQNTPIGDGPVLLPDNHYLDTHSALSKDPNEKRDHMVLLEFVTAAGITHGMDELY
KLEHHHHHHC

pre-SufI(IAC)
(plasmid p-preSufI(IAC); *Addgene #168516*):

MSLSRRQFIQASGIALIAGAVPLKASAAGQQQPLPVPPLLESRRGQPLFMTVQRAHWSFTPGTRASVWGI
NGRYLGPTIRVWKGDDVKLIYSNRLTENVSMTVAGLQVPGPLMGGPARMMSPNADWAPVLPIRQNAATLW
YHANTPNRTAQQVYNGLAGMWLVEDEVSKSLPIPNHYGVDDFPVIIQDKRLDNFGTPEYNEPGSGGFVGD
TLLVNGVQSPYVEVSRGWVRLRLLNASNSRRYQLQMSDGRPLHVISGDQGFLPAPVSVKQLSLAPGERRE
ILVDMSNGDEVSITAGEAASIVDRIRGFFEPSSILVSTLVLTLRPTGLLPLVTDSLPMRLLPTEIMAGSP
IRSRDISLGDDPGINGQLWDVNRIDVTAQQGTWERWTVRADEPQAFHIEGVMFQIRNVNGAMPFPEDRGW
KDTVWVDGQVELLVYFGQPSWAHFPFYFNSQTLEMADRGSIGQLLVNPVPLEIKRASQPELAPEDPEDVE
HHHHHHC

Fig S1. Protein sequences for the purified proteins used in this study. The signal peptides of TorA and SufI are underlined, the TEV sequence is identified in *blue*, the QSV tag is identified in *green*, and other additions/linkers/mutations are indicated in *red*.
